# Effects of SAFE Early Intervention Approach in the First Months of Life in Infants at Risk: A Randomized Controlled Study

**DOI:** 10.1111/cch.70107

**Published:** 2025-05-28

**Authors:** Ayse Yildiz, Ramazan Yildiz, Umut Apaydin, Pelin Atalan Efkere, Kivilcim Gücüyener, Ibrahim Murat Hirfanoglu, Bulent Elbasan

**Affiliations:** ^1^ Department of Physical Therapy and Rehabilitation, Faculty of Health Sciences Erzurum Technical University Erzurum Turkey; ^2^ Department of Physiotherapy and Rehabilitation, Faculty of Health Sciences Karadeniz Technical University Trabzon Turkey; ^3^ Department of Physical Therapy and Rehabilitation, Faculty of Health Sciences Gazi University Ankara Turkey; ^4^ Department of Pediatric Neurology Gazi University Faculty of Medicine Ankara Turkey; ^5^ Department of Neonatology Gazi University Faculty of Medicine Ankara Turkey

**Keywords:** early intervention, enriched environment, infants at risk, physiotherapy

## Abstract

**Background:**

This study aimed to examine the effects of the SAFE early intervention approach (Sensory strategies, Activity‐based motor training, Family collaboration, and Environmental Enrichment), developed for at‐risk infants, on motor, cognitive, language development, and sensory processing skills in the first 3 months of life.

**Methods:**

Twenty‐six infants with a corrected age of 42 weeks were included in the study, and the infants were randomly distributed to the treatment and control groups. The SAFE early intervention approach was applied to 14 infants in the treatment group, and the Neurodevelopmental Treatment‐based home programme was applied to 12 infants in the control group. All infants included in the study were evaluated at the corrected 42nd week before the intervention and at the corrected 12th week after the intervention by an evaluator blinded to the treatment. Bayley Scales of Infant and Toddler Development III (Bayley‐III) to evaluate cognitive and motor development; Test of Infant Motor Performance (TIMP) to evaluate neuromotor development; Infant/Toddler Sensory Profile 2 (ITSP 2) to assess sensory processing t was used. The Infant/Toddler HOME Inventory was used to evaluate the home environment.

**Results:**

The interaction effects (time × group) revealed significant advantages for the SAFE early intervention group, evidenced by higher scores in the Bayley‐III motor composite, TIMP elicited and HOME total assessments (*p* < 0.05). However, the interaction effects (time × group) showed no differences between the groups in the Bayley‐III cognitive and language composite scores, as well as the TIMP observed and TIMP total scores (*p* > 0.05). The main effect for time was significant in all parameters (*p* < 0.05). Similarly, the main impact for groups was substantial in all evaluation parameters except the Bayley‐III language composite score and TIMP observed score (*p* < 0.05). The interaction effects (time × group) demonstrated significant differences in favour of the SAFE early intervention group for the general processing score, auditory processing score, tactile processing score and total score of the ITSP 2 (*p* < 0.05).

**Conclusions:**

The SAFE early intervention approach enhanced motor and sensory outcomes and provided a more enriched home environment than the NDT‐based home programme. It was concluded that neurodevelopmental improvement will be achieved with the SAFE early intervention approach in the early period in at‐risk infants.

**Trial Registration:** NCT06361134

**Summary**

- The SAFE intervention approach provides an enriched home environment
- The SAFE approach increases early motor development.
- Family collaboration increases early intervention success.
- An enriched home environment supports infant development.
- SAFE intervention positively affects general, tactile, and auditory processing skills

## 1 Introduction

At‐risk infants are defined as infants who are exposed to situations that may cause developmental problems and have higher‐than‐average mortality and morbidity. These infants may have developmental delays due to maternal factors and difficulties encountered during pregnancy and birth (Formiga et al. 2018).

Early intervention approaches have been implemented in many countries for decades to improve the developmental outcomes of at‐risk infants and the family's well‐being. In Turkey, services for infants with special needs and their families are provided ‘centre‐based’ and accompanied by a physiotherapist. However, early intervention approaches in natural settings (home environments) support children's development more effectively than traditional intervention models in clinical offices or centres (Raab and Dunst 2004). Natural environments provide environments for children to learn behaviours identified as necessary by adults and support child‐initiated activities in daily activities. Additionally, collaboration with the family positively impacts parent involvement and intervention outcomes, including the performance of functional tasks, the child's developmental gains, parent satisfaction with health services, increased goal attainment and parents' psychological well‐being (Law et al. 1998). In recent years, the importance of early intervention approaches carried out with the participation of families has been increasing in the literature. The Goal, Activity, Motor Enrichment (GAME), in which children collaborate with the therapist by setting goals for their development and achieving the determined goals in the home environment, has been accepted in recent years (Löwing et al. 2009). In this context, the routine‐based early intervention method argues that routines in natural environments for young children are most effective in supporting and sustaining early intervention activities (Vygotsky 1933). With the COPCA approach, another method that adopts the family‐centred early intervention approach, the physiotherapist trains the family to recognize the infant's signals and respond appropriately to the infant's real needs (Dirks et al. 2011). In Neurodevelopmental Treatment (NDT), the most widely used method in our country within the scope of early intervention methods, it is essential to support and educate parents and include them in the treatment programme, considering the family's needs. The family plans the treatment programme, carries out treatment activities and sets goals for the home environment. Still, the key person in the intervention process is the therapist, and the intervention is child centred (Bly 1999; Howle 2002). However, children learn within the context of their families, which primarily influences their learning and development. For this reason, adequate cooperation with the family cannot be achieved with the early intervention approaches implemented in our country. Therefore, the optimal participation of the child and the family in the treatment process remains limited.

Early intervention approaches focusing on family factors and the home environment after hospital discharge significantly impact long‐term morbidity. Due to the significant impact of environmental and social factors on the development of children, particularly those at high risk, the importance of environmental enrichment programmes has grown in recent years. These programmes aim to enhance cognitive or motor outcomes by providing an optimal learning environment (Shonkoff 2003). Because infants' voluntary access to their environment occurs long after birth, they depend on their parents for access to an enriched environment (Morgan et al. 2013). At this point, it is essential for intervention programmes that parents provide ‘affordance’, giving action opportunities to infants with objects, events or places in the environment. In this way, the baby can explore new environments and learn by trial and error.

Early intervention approaches aim to optimize motor development, but the theoretical foundations of these programmes differ. Most physiotherapy and rehabilitation interventions are based on Neurodevelopmental Treatment (NDT) principles, which focus on modifying sensory input and abnormal movement patterns through active and passive techniques (Blauw‐Hospers and Hadders‐Algra 2005). Although NDT has been widely used, it often limits the infant's movement exploration and motor learning opportunities. In contrast, approaches that provide just‐right challenges in varied contexts encourage the infant to explore and develop a broader motor repertoire (Dirks et al. 2011; Hadders‐Algra 2000). Despite the widespread use of NDT, the evidence supporting its effectiveness remains controversial. Some systematic reviews and meta‐analyses have reported limited or inconsistent benefits of NDT compared with more activity‐based and function‐focused interventions (Brown and Burns 2001; Te Velde et al. 2022). Therefore, developing an intervention approach that excludes passive treatments and focuses on challenging the child with sensory and motor strategies is necessary. In this context, the SAFE early intervention approach was developed by the Gazi University Faculty of Health Sciences Pediatric Physiotherapy and Rehabilitation department, and it was shown to improve cognitive, speech, and language development and sensory outcomes in preterm infants with a corrected age of 9–10 months (Apaydın et al. 2023). This study aimed to examine the effects of the SAFE early intervention approach in at‐risk infants' first 3 months of life on motor, sensory, cognitive and language development.

## 2 Methods

### 2.1 Participants

This study was conducted in the paediatric rehabilitation unit of Gazi University Faculty of Health Sciences between January 2021 and June 2022. We calculated the sample size (at least 12 infants in each group) using a power analysis program (G*power version 3.1.9.2, Axel Buchner, Universität Kiel). The power is set at 0.8 and alpha at 0.05 (Morgan et al. 2014). The study was completed with 26 infants (SAFE, *n* = 14, NDT, *n* = 12). Postpower analysis revealed a total of 26 patients with the *α* = 0.05 and *η* ^2^ = 0.313 for primary outcome measure corresponds to Power (1 − β err prob) = 0.8647656. Detailed oral and written information was given to the parents of all infants. The Gazi University Clinical Research Ethics Committee received permission to conduct the study (14574941‐302.08.01‐).

Infants at risk of cerebral palsy (CP) or other neurodevelopmental disorders after birth were included in the study when their corrected age was 2 weeks after discharge from the neonatal intensive care unit (NICU). The infants included in the study were diagnosed as risk infants by a paediatric neurologist (K.G). Criteria for inclusion in the study: being diagnosed with a neurologically and developmentally at‐risk infant (such as premature birth, hypoxia, infection, hypoxic–ischaemic encephalopathy), having a NICU history of 15 days or more, having completed their medical treatment and having terminated NICU treatment at the 42nd week of pregnancy at the latest. Infants with congenital malformations, metabolic and genetic diseases, musculoskeletal anomalies and those on a ventilator were excluded from the study. Parents of eligible infants were informed about the study after an interview regarding the infant's risk status. Voluntary consent for the study was obtained from the families. Infants of parents who did not wish to participate in the study continued to receive usual care.

### 2.2 Study Protocol

**FIGURE 1:**
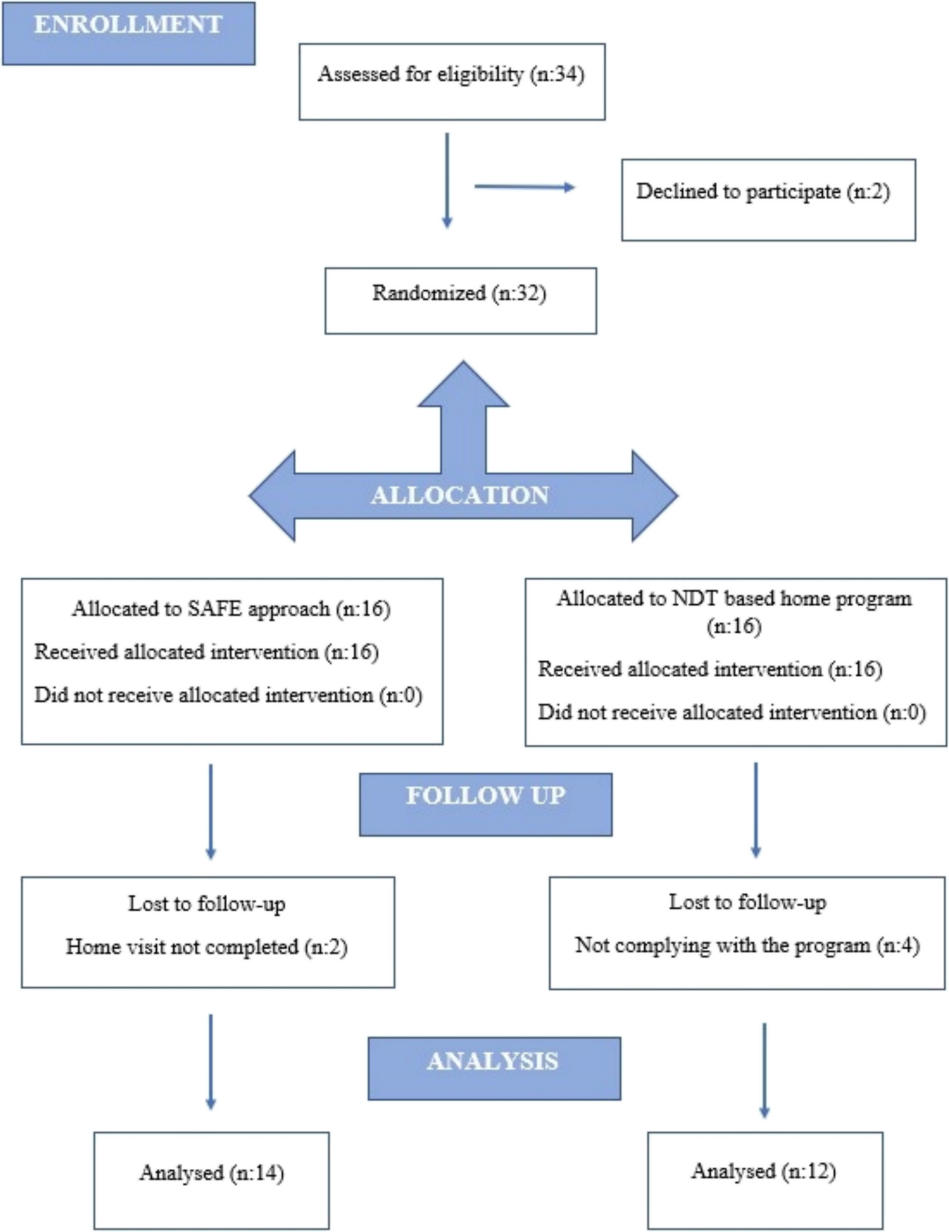
CONSORT flowchart showing.

This study was designed as a randomized, controlled and single‐blinded study. Thirty‐two infants were eligible to participate in the study. Block randomization assigned participants to the SAFE intervention group (*n* = 16) and the NDT group (*n* = 16). Randomization was based on a prepared random assignment number list kept in a locked cabinet by the principal investigator. The researcher who performed the randomization was not involved in the data collection or evaluation process. The two researchers who collected the data and made the evaluations needed to be made aware of which group the children were in. All assessments and interventions were performed by therapists with an average of 10 years of experience in paediatric rehabilitation. Figure 1 shows the study design with the Consolidated Standards of Reporting Trials (CONSORT) flowchart (Figure 1).

### 2.3 Intervention

SAFE Early Intervention: The SAFE approach embraces family collaboration and focuses on activity‐based motor learning and sensory strategies in an enriched environment. The initials of the principles on which it is based form the name of the programme (S: Sensory Strategies, A: Activity Based Motor Training, F: Family Collaboration, E: Environmental Enrichment). The theoretical framework of the approach consists of motor learning principles and neuronal group selection theory (NGST). Within the scope of NGST, facilitation of movements through hand contact techniques is avoided because these techniques interfere with the infant's activity and motor learning processes.

During the intervention, the infant could explore their daily activities with the right challenges under various conditions. Environments that enable the child to be active and explore are created, and plenty of active trial and error exercises are included in multiple conditions. The motor learning process was supported with ‘hands‐off’ methods. The intervention was carried out in the child's home environment, and parent–infant interaction was supported from an early age by the physiotherapist coaching the parents to implement the programme. The SAFE early intervention approach has a multidimensional structure to support social–emotional, motor, sensory and communication development. The sensory dimension of the approach aims to regulate the infant's responses to environmental stimuli, balance the level of alertness and support sensory integration skills. During the intervention, the infants' reactions to visual, auditory and tactile stimuli were observed, and parents were given guidance on how to understand these sensory responses and support them appropriately. After the first evaluation, the family was given a booklet on helping the baby's sensorimotor development, and the information and applications in the brochure were practically demonstrated. Families were asked to do these activities every day for 10 weeks and activities that supported their neuromotor development that overlapped with these activities within their daily routines. The family and caregivers explained in detail that interaction and communication were the main elements during the activities. The first home visit was made 15 days after the initial assessment, and the home environment was evaluated. Home visits were planned monthly, and at least three visits were organized. The intervention programme was planned to last 12 weeks. To ensure follow‐up of the intervention, a phone call was made to the family once a week, and the family members were asked to keep a diary.

NDT‐based home programme: NDT principles, which are routinely used in physiotherapy and rehabilitation practices in our country, were applied to the infants in the control group. Following the initial evaluation, an NDT‐based treatment programme was created and taught to the family. The family was asked to implement this programme at home. The treatment movements were shown to the mother in practice; they were told how to make hand contact and that they should apply the treatment programme for 30–45 min a day, 7 days a week, depending on the child's tolerance. Phone calls were made to ensure the treatment was progressing correctly. One month after the first evaluation, the infants were called to the clinic again for examination. After the assessment, the treatment programmes were rearranged to suit their motor development stages. The infants' examinations were completed monthly in the clinical environment, and new treatment programmes were created. The infants' home environments in the control group were evaluated in two visits before and after the intervention.

### 2.4 Measurements

Demographic information on the infants was recorded during the first interview. The initial assessment was made during the corrected 42nd week, and the last evaluation was made during the corrected 12th week at the Gazi University Faculty of Health Sciences Pediatric Rehabilitation clinic. The home environment was assessed by observing the parent's attitude and environment during the home visit for the SAFE intervention group. The control group received two visits to evaluate the home environment before and after the intervention.

### 2.5 Neuromotor Development

Test of Infant Motor Profile (TIMP) assessed functional motor behaviour (Campbell and Hedeker 2001). TIMP is used to identify infants with delayed motor development, to differentiate between at‐risk infants with poor motor development and to measure change resulting from intervention. It evaluates the postural and selective movement control required for functional motor performance in early infancy. The test consists of a total of 42 items. The first 13 items (observed items) involve observation of spontaneous movement; Movements such as the midline of the head, reaching, and independent finger movements are scored as ‘yes‐no’. Then, the baby is placed in different positions, stimulated with images and sounds, and the answers given are scored on an ordinal scale of 3, 4, 5 or 6 points (elicited items) (Campbell and Hedeker 2001).

### 2.6 Motor, Cognitive and Language Development

Cognitive, language and motor subscales of Bayley Scales of Infant and Toddler Development III (Bayley‐III), considered the gold standard for evaluating general development, were used. The child receives 0 or 1 point for each item, depending on whether the child meets the scoring criteria. Raw scores are summed for each subtest (total Raw Score). It is then converted into scale scores, composite scores, percentile ranks, and confidence intervals using the tables provided in the manual (Tecklin 2008).

### 2.7 Sensory Processing Skills

The Infant/Toddler Sensory Profile 2 (ITSP 2), completed by the primary caregiver, was used to evaluate the sensory processing abilities of infants. Parents rate the child's behaviour frequency on a five‐point scale from 1 (*almost always*) to 5 (*almost never*). Items on this test evaluate general, auditory, visual, tactile, vestibular and oral sensory processing ability. A high score indicates infants notice and respond to stimuli less than their peers, meaning they are hypersensitive. In comparison, a low score suggests infants notice and react to stimuli more than their peers or are hypersensitive (Dunn 2002).

### 2.8 Home Environment

The Infant/Toddler HOME Inventory (HOME) is widely used worldwide to assess many aspects of the home environment that are thought to be influential in children's development (Bradley and Caldwell 1984). The scale comprises 45 items and six subscales (Responsivity, Acceptance, Organization, Learning Materials, Involvement and Variety). Each statement on the scale is scored as yes or no. A high total score indicates an enriched home environment (Totsika and Sylva 2004).

### 2.9 Statistical Analysis

The SPSS program 26 (SPSS Inc. Chicago, IL, USA) was used for data analysis. Data normality was evaluated Using visual and analytical methods—such as histograms and the Shapiro–Wilk test. T1 (pre‐treatment) data were compared across groups using either a Mann–Whitney *U* test or an independent samples *t* test. The T1 and T2 (posttreatment) findings within groups were compared using the Wilcoxon rank test or the paired samples *t* test. Cohen's norms were used to calculate the effect sizes for within‐group T1–T2 variations. Small effect (0.2), moderate impact (0.5) or substantial effect (0.8) were the reported Cohen's *d* findings. Two‐way repeated‐measures ANOVA was used to investigate the time × group interaction. Partial eta‐square (*ηp* ^2^) was interpreted as effect size as small (*ηp* ^2^ = 0.01), medium (*ηp* ^2^ = 0.06) and large (*ηp* ^2^ = 0.14) (Jacob 1988).

## 3 Results

The study was completed with 26 infants, 14 in the SAFE intervention group and 12 in the NDT group. There was no difference between the groups in terms of demographic characteristics of the infants included in the study (*p* > 0.05) (Table 1). Risk factor information for infants is given in Table 2. There was no difference between the groups regarding risk factors (*p* > 0.05).

**TABLE 1:** Demographic characteristics of infants.

Infants characteristics	SAFE (*n* = 14) Median (IQR)	NDT (*n* = 12) Median (IQR)	p*
Gestational age (week)	30.5 (28–36.75)	33 (29–38.75)	0.51
Birth size (cm)	47 (37.75–49)	41 (29–38.75)	0.77
Birth weight (g)	1650 (1200–2500)	1530 (1290–3300)	0.47
NICU stay (days)	30 (7–64)	32.5 (6.50–53)	0.58

	*n* (%)	*n* (%)	p**
Sex			
Female	7 (50)	5 (41.70)	0.67
Male	7 (50)	7 (58.30)	
Birth			
Preterm	11 (78.60)	9(75)	0.82
Term	3 (21.40)	3 (25)	
Mother's education			
Secondary school	3 (21.40)	4 (33.30)	0.47
High school	3 (21.40)	4 (33.30)	
Bachelor degree	8 (57.10)	4 (33.30)	

Note: p* Mann–Whitney *U* test, p** chi‐square test or Fisher's exact test; *p* < 0.05.

Abbreviations: IQR: interquartile range, NICU: neonatal intensive care unit, NDT: Neurodevelopmental Treatment‐based home programme, SAFE: SAFE Early Intervention Approach.

**TABLE 2:** Risk factors for infants.

Risk factors	SAFE (*n* = 14) *n* (%)	NDT (*n* = 12) *n* (%)	*p*
Respiratory distress syndrome	3 (21.40)	3 (25)	0.56
Hypoxic ischaemic encephalopathy	3 (21.40)	4 (33.30)	
Intraventricular haemorrhage	4 (28.50)	4 (33.30)	
Intrauterine growth retardation	2 (14.20)	1 (8.30)	
Periventricular leukomalacia	2 (14.20)	0	

*Note:* Pearson chi‐square test, *p* < 0.05.

Abbreviations: NDT: Neurodevelopmental Treatment‐based home programme; SAFE: SAFE Early Intervention Approach.

As a result of the intervention, interaction effects (time × group) showed significant differences in favour of the SAFE early intervention group for Bayley‐III motor composite score, TIMP elicited score, and HOME total score (*p* < 0.05) (Table 3).

**TABLE 3:** The Bayley‐III, TIMP and HOME scores of infants.

Outcome	Group	T1 mean ± SD	T2 mean ± SD	Change mean ± SD	Cohen's *d*	The main effect for group *p* *F* *ηp* ^2^	The main effect for the time *p* *F* *ηp* ^2^	Time × group *p* *F*	*ηp* ^2^
Bayley‐III cognitive composite score	SAFE	90 ± 14.93	122.85 ± 15.89	32.85 ± 18.78	0.57	**0.03** **5.12** **0.17**	**< 0.001** **68.21** **0.74**	0.36 0.85	0.14
	NDT	83.75 ± 12.99	110 ± 11.48	26.25 ± 17.46	0.66				
Bayley‐III language composite score	SAFE	83.78 ± 32.07	119.42 ± 17.10	35.64 ± 30.35	0.85	0.42 0.65 0.02	**< 0.001** **28.73** **0.54**	0.05 3.92	0.14
	NDT	87.83 ± 14. 40	104.25 ± 15.14	16.41 ± 15.51	0.94				
Bayley‐III motor composite score	SAFE	85.42 ± 5.86	114.14 ± 13.89	28.71 ± 14.46	0.50	**0.02** **5.8** **0.19**	**< 0.001** **74.681** **0.75**	**0.003** **10.91**	0.31
	NDT	86.25 ± 5.02	99.08 ± 10.71	12.83 ± 8.87	0.69				
TIMP observed	SAFE	25.08 ± 8.98	92.62 ± 16.53	67.53 ± 20.01	0.29	0.06 3.66 0.14	**< 0.001** **212.55** **0.91**	0.20 1.71	0.07
	NDT	22.80 ± 12.86	79.20 ± 16.78	56.40 ± 20.46	0.36				
TIMP elicited	SAFE	7.54 ± 1.66	10.38 ± 1.98	2.84 ± 2.19	0.77	**0.04** **4.76** **0.18**	**0.008** **8.45** **0.28**	**0.008** **8.45**	0.28
	NDT	7.50 ± 1.17	7.50 ± 2.79	0 ± 2.49	0				
TIMP total	SAFE	32.62 ± 8.94	103 ± 16.90	70.38 ± 20.60	0.29	**0.03** **5.06** **0.19**	**< 0.001** **212.38** **0.91**	0.12 2.58	0.11
	NDT	30.30 ± 12.82	86.70 ± 17.28	56.40 ± 20.78	0.36				
HOME total score	SAFE	29.77 ± 5.44	39.92 ± 6.07	10.15 ± 5.88	0.57	**0.01** **7.45** **0.24**	**< 0.001** **70.62** **0.75**	**0.005** **9.68**	0.29
	NDT	28.33 ± 2.34	33 ± 1.80	4.66 ± 1.66	0.35				

*Note: p* < 0.05 for interaction (time × group) by analysis of variance (ANOVA). T1: pretreatment, T2: posttreatment. Statistically significant results (*p* < 0.05) are highlighted in bold.

Abbreviations: HOME: Infant/Toddler HOME Inventory, NDT: Neurodevelopmental Treatment‐based home programme, SAFE: SAFE Early Intervention Approach, SD: standard deviation, TIMP: Test of Infant Motor Profile.

**FIGURE 2:**
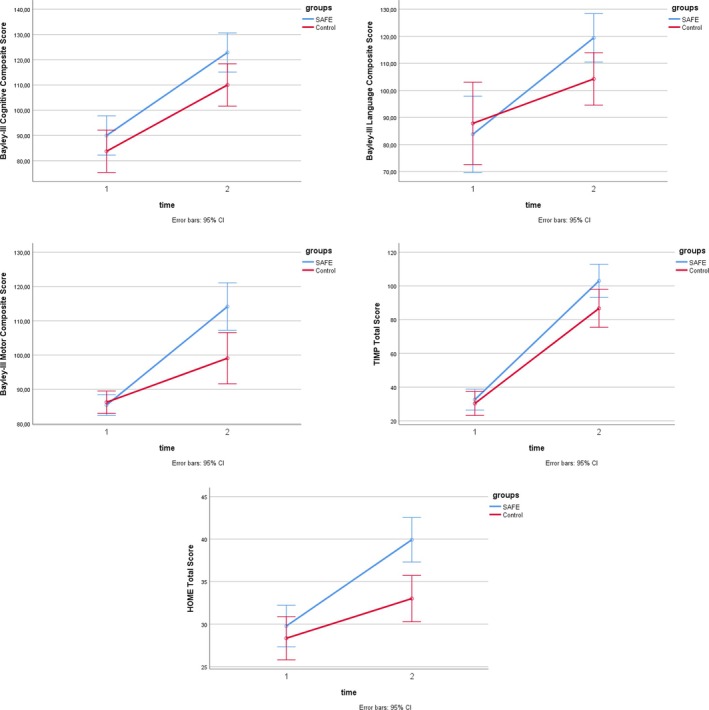
Comparisons of treatment and control groups.

In pairwise comparisons, we examined the groups within themselves pre‐ and post‐intervention (SAFE group: T2 vs. T1, NDT group: T2 vs. T1). We also compared the pre‐and post‐intervention situations between groups (T1: SAFE vs. NDT group, and T2: SAFE vs. NDT group). Bayley‐III cognitive score comparisons were, respectively, SAFE group: T2 versus T1 (*p* = 0.002), NDT group: T2 versus T1 (*p* = 0.003), T1: *p* = 0.33, and T2: *p* = 0.07. Bayley‐III language score comparisons were, respectively, SAFE group *p* = 0.001, NDT group *p* = 0.004, T1: *p* = 0.48, and T2: *p* = 0.03. Bayley‐III motor score comparisons were SAFE group *p* = 0.001, NDT group *p* = 0.003, T1: *p* = 0.87, and T2: *p* = 0.005. TIMP total score comparisons were, respectively, SAFE group *p* = 0.001, NDT group *p* = 0.04, T1: *p* = 0.34, and T2: *p* = 0.04. HOME total score comparisons were SAFE group *p* = 0.001, NDT group *p* = 0.002, T1: *p* = 0.32, and T2: *p* = 0.001 (Figure 2).

Despite these improvements, there was no difference in the interaction effects (time × group) of the groups in terms of the Bayley‐III Cognitive and Language score, TIMP Observed and TIMP Total score (*p* > 0.05) (Table 3).

In terms of sensory processing, the interaction effects (time × group) revealed significant differences in favour of the SAFE group for the general raw score, auditory raw score, tactile raw score and total score of the ITSP‐2 (*p* < 0.05) (Table 4). However, no significant interaction was found for the visual, movement or oral raw scores (*p* > 0.05). A significant main effect of time was observed in the auditory and oral raw scores (*p* < 0.05). In contrast, no significant main effect of the group was detected in any of the ITSP‐2 parameters (*p* > 0.05) (Table 4).

**TABLE 4:** ITSP scores of infants.

Outcome	Group	T1 mean ± SD	T2 mean ± SD	Change mean ± SD	Cohen's *d*	The main effect for group *p* *F* *ηp* ^2^	The main effect for the time *p* *F* *ηp* ^2^	Time × group *p* *F*	Time × group *ηp* ^2^
ITSP 2 general processing	SAFE	14.60 ± 3.60	19.10 ± 3.90	4.50 ± 3.98	1.13	0.48 0.51 0.02	0.43 0.63 0.02	**0.001** **15.95**	**0.43**
	NDT	17.30 ± 4.40	14.30 ± 4.60	−3 ± 5	0.6				
ITSP 2 auditory processing score	SAFE	8.80 ± 2.20	12.30 ± 1.50	3.50 ± 2.46	1.42	0.43 0.62 0.02	**0.003** **11.23** **0.34**	**0.03** **5.38**	**0.20**
	NDT	11 ± 4	11.60 ± 2.10	0.60 ± 3.40	0.17				
ITSP 2 visual processing score	SAFE	6.30 ± 3.20	8.40 ± 2.30	2.08 ± 3.52	0.59	0.65 0.20 0.01	0.19 1.78 0.07	0.05 4321	0.17
	NDT	7 ± 3.10	6.60 ± 3.40	−0.40 ± 2.06	0.19				
ITSP 2 tactile processing score	SAFE	4.2 ± 2.20	6.80 ± 2.40	2.50 ± 3.20	0.78	0.09 3.10 0.12	0.12 2.63 0.11	**0.005** **9.78**	**0.31**
	NDT	4.50 ± 2.80	3.70 ± 1.60	−0.80 ± 1.70	0.47				
ITSP 2 vestibular processing score	SAFE	10 ± 2.10	10.20 ± 3.30	0.16 ± 4.50	0.03	0.47 0.53 0.02	0.68 0.17 0.008	0.53 0.39	0.01
	NDT	9.80 ± 3.20	9 ± 3.50	−0.80 ± 2.50	0.32				
ITSP 2 oral processing score	SAFE	3.20 ± 1.20	5.40 ± 0.60	2.10 ± 1.26	1.66	0.74 0.10 0.00	**< 0.001** **41.294** **0.66**	0.38 0.80	0.03
	NDT	3.60 ± 1.40	5.20 ± 1	1.60 ± 1.50	1.06				
ITSP 2 total score	SAFE	47.20 ± 8.40	62.20 ± 8.80	15 ± 7.10	2.11	0.35 0.91 0.04	0.11 2.74 0.11	**< 0.001** **23.55**	**0.52**
	Control	54.70 ± 12.60	47.30 ± 12.70	−7.3 ± 14.10	0.51				

*Note: p* < 0.05 for interaction (time × group) by analysis of variance (ANOVA). Statistically significant results (*p* < 0.05) are highlighted in bold.

Abbreviations: ITSP 2: Infant/Toddler Sensory Profile 2, NDT: Neurodevelopmental Treatment‐based home programme, SAFE: SAFE Early Intervention Approach, SD: standard deviation.

## 4 Discussion

This study showed a significant group‐by‐time interaction in favour of the SAFE group regarding at‐risk infants' motor and sensory processing skills. Additionally, the intervention provided an enriched home environment.

Many countries have developed national‐level early intervention programmes tailored to their specific needs and available resources (Morgan et al. 2014; Finlayson et al. 2020). Considering the importance of cultural differences, developing country‐specific approaches is essential for the effectiveness and applicability of early intervention strategies. In our country, the SAFE early intervention approach represents the first structured programme designed specifically for at‐risk infants—those who are exposed to factors that may lead to developmental delays and have higher‐than‐average risks of mortality and morbidity. This approach is intended for use beginning early following NICU discharge, offering timely support for the infant and their family. Unlike many models in the literature, SAFE includes sensory strategies to support sensory development, offering a comprehensive alternative within early physiotherapy and rehabilitation practices in our national context.

Early intervention approaches are not designed to provide high‐intensity, high‐dose intervention during maximum neuroplasticity in the first month of life (Atkins et al. 2017). However, it is known that most preterm infants only benefit from the early intervention programme once they are corrected to 3 months old (Little et al. 2015). Due to this delay, most infants cannot receive intervention during the critical period for plasticity. This also means that at‐risk infants will only benefit from early intervention once a significant delay is documented or CP is diagnosed. In this study, with the SAFE early intervention approach, intensive intervention was aimed at the earliest period during the transition to home after NICU discharge.

Several interventions are aimed at improving neurodevelopmental outcomes in at‐risk infants. Finlayson et al. applied the SPEEDI approach, emphasizing the importance of early intervention until the infants' corrected age is 3 months. As a result of the study, it was found that there was an improvement in language and gross motor development in the 4th month (Finlayson et al. 2020). Hielkema et al. conducted a randomized controlled trial that included infants with abnormal GMs with a corrected age of 3 months. They found that the COPCA method did not make a difference in motor development (Hielkema et al. 2011). In the study by Ziegler et al., the COPCA method and standard care were compared in preterm infants born before 32 weeks. The study results found that the motor development of infants in the COPCA group was better at the 18th month. Still, there was no difference between the groups regarding cognitive and motor development in the 24th month (Akhbari Ziegler et al. 2021). Although the COPCA approach encourages diversity in motor behaviour and trial‐and‐error experiences, it ignores the need to provide an enriched environment for motor learning. However, the benefits of environmental enrichment on brain recovery have been proven in the literature (Novak et al. 2013). The GAME method, developed in 2015, adopts target‐oriented activity‐based therapy approaches and provides optimal environments for motor learning by providing an enriched environment. Studies using the GAME approach have shown that it increases motor and cognitive development (Morgan et al. 2014, 2015). Apaydin et al. found that the SAFE approach improved cognitive, speech‐language, and sensory outcomes compared with the NDT‐based home programme (Apaydın et al. 2023). Considering the effects of environmental enrichment on development, our study focused on creating a sensory, motor, and psychomotor‐enriched home environment. Infants were allowed to explore with ‘hands‐off’ techniques in an enriched environment, making treatment a part of the daily routine. Reducing hand contact helped the child uncover many skills through trial and error. All these factors positively affected the motor development of infants. The fact that GAME therapy, which is based on the principle of environmental enrichment, also increases motor development supports the positive effect of the enriched environment on motor development. Additionally, as a result of the study, there was an increase in TIMP motor scores in favour of the SAFE intervention group, strengthening our finding that the intervention increased motor development.

It is generally accepted that at‐risk infants have lower cognitive scores than typically developing infants and that their cognitive abilities may deteriorate over time. Therefore, early intervention programmes often aim to improve motor outcomes and facilitate cognitive development (Bredy et al. 2003). Recent evidence suggests that early intervention positively impacts the cognitive outcomes of preterm infants, regardless of the type of therapy. Any form of early intervention is linked to enhanced cognitive function at 1–2 years of age (McManus and Rosenberg 2012). In line with this evidence, both interventions improved cognitive development in this study, but no difference was found between the groups. The evaluation made in a short period, such as 3 months, may not have shown the effectiveness of the intervention on cognitive development; it may have effects on cognitive development in the long term (1–2 years). Therefore, a long‐term follow‐up study is necessary.

There are studies in the literature investigating the effects of enriched environmental interventions on at‐risk infants with brain damage and children older than 1 year old with a diagnosis of CP. In the scant literature on the benefits of environmental enrichment for at‐risk infants, premature infants have been shown to gain neurobehavioural improvements from sensory‐specific environmental enrichment activities such as massage and music (Guzzetta et al. 2009; Lubetzky et al. 2010). As a result of the study, a significant difference was observed in favour of the SAFE intervention group in the HOME Inventory total score. With the SAFE approach, parent‐infant interaction is supported, and passive communication methods are limited to create environmental richness in the early period. Multisensory interventions were ensured to be implemented in the daily routine, sufficient play materials were provided, and toys/books/educational cards were requested to be used and positioned in the home in a way that would support development. The interventions created an environment enriched in motor terms and sensory aspects, thus helping motor and sensory development. Therefore, at‐risk infants who have negative sensory experiences due to a NICU history should be supported in a sensory and motor‐enriched environment, and the family should be encouraged in this regard.

In at‐risk infants, excessive sensory stimuli and multiple painful procedures in the NICU can disrupt the infant's brain development. Stress can affect the infant's multisensory integration processes and perception of self and environment and can lead to negative consequences on development (Als et al. 2004). It has been reported that especially preterm and low birth‐weight infants are at risk in terms of sensory development (Kara et al. 2020; Chorna et al. 2014). When sensory integration therapy‐based studies in the literature were examined, it was seen that sensory‐based interventions improved sensory processing skills. Pekçetin et al. applied 8‐week sensory integration therapy to preterm infants with a corrected age of 7 months. They showed an improvement in the sensory processing scores of preterm infants related to tactile, deep pressure, vestibular and visual systems, and adaptive motor functions (Pekçetin et al. 2016). Lecuona et al. applied 10 sessions (once a week for 10 weeks) of sensory integration intervention to 12 preterm infants with corrected ages between 4 and 10 months. They significantly reduced sensory processing disorder in preterm infants compared with the control group (Lecuona 2012). No systematic early intervention programme was developed to support sensory development for at‐risk infants from the early period after discharge from the hospital. While early intervention programmes developed in recent years have focused more on the principles of enriched environment and motor learning, they have ignored practices to support sensory development (Dirks et al. 2011; Morgan et al. 2014). Therefore, the SAFE early intervention programme aimed to fill this gap in the literature. Within this study, multisensory interventions were applied by parents and caregivers as part of the daily routine in a sensory, cognitive and psychomotor‐enriched home environment to support infants' sensory development. The study found a significant difference in favour of the SAFE treatment group regarding ITSP 2 general processing, tactile processing, auditory processing skill scores, and total ITSP 2 score. It was thought that the infants in the treatment group could tolerate sensory stimuli better because of the treatment; they were more aware of their environment and the possible sensory input it contained, and their sensory seeking decreased. As a result, their motor responses would be better.

A limitation of the study is that the inclusion criteria are not limited and include all risk groups. Examining the effectiveness of the SAFE approach in groups with specific risk factors, such as ‘early preterm infants with brain damage’, may increase the quality of the study. Additionally, there is a need to investigate whether ‘hands‐off’ techniques will be equally effective when applied to groups at high risk of CP. This study shows the effectiveness of the intervention used in infants in the first 3 months of life. Still, it is essential to follow the long‐term results of the intervention to evaluate its effect on neurodevelopmental outcomes.

## 5 Conclusions

Our study shows that the SAFE early intervention approach is more effective than the NDT approach in improving the motor and sensory functions of at‐risk infants aged 0–3 months in an enriched home environment. The SAFE approach is a promising intervention that supports the neurodevelopmental outcomes of at‐risk infants in the early period.

## Author Contributions

**Ayse Yildiz:** project administration, conceptualization, formal analysis, investigation, methodology, writing – original draft, writing. **Ramazan Yildiz:** investigation, methodology, writing. **Umut Apaydin:** formal analysis, methodology, writing. **Pelin Atalan Efkere:** investigation, methodology. **Kivilcim Gücüyener:** project administration, methodology. **Ibrahim Murat Hirfanoglu:** methodology. **Bulent Elbasan:** project administration, review, and editing.

## Ethics Statement

The Gazi University Clinical Research Ethics Committee received permission to conduct the study (14 574941‐302.08.01‐). Parents were asked to sign a consent form to allow their infants to participate in the study.

## Conflicts of Interest

The authors declare no conflicts of interest.

## Data Availability

The datasets used and analysed during the current study are available from the corresponding author upon reasonable request.
